# Gloucester General Infirmary

**Published:** 1832-02

**Authors:** 


### GLOUCESTER GENERAL INFIRMARY.

*Case of Peritonitis, excited by long-continued Attempts to find the Stone with the Staff before Operation. Two Stones; one sacculated; a Piece left in that situation safely.**

*Garret Ward, Infirmary.* I performed lithotomy, February 19, 1817, upon — Holiday, of Nailsworth, a man seventy-three years of age, but of good constitution. There was a considerable difficulty in feeling the stone with the staff, and attempts were indiscreetly made by many surgeons who were present, for a considerable time, so that a fear was expressed that injury might be done to the bladder.

There was nothing remarkable observed in the operation, excepting that, besides a large stone, the bladder also contained a smaller one encysted. I felt it projecting immediately above the pubis, where it was evidently firmly fixed. A long pair of dressing forceps broke off the point in the attempt to extract it from its bed, and the remainder was left behind, which the nail of the fore-finger could plainly distinguish to be on a level with the sides of the cyst and the bladder.

No further attempts were made to dislodge the stone. The severity of the sounding, which led to this decision, was not forgotten. It was not desirable to add more irritation to what had been already endured, and the stone was permitted to remain undisturbed in its habitation.

In the night, after the operation, he had severe pain just above the pubis, where was likewise some swelling; his skin was rather hot, the tongue somewhat furred. Pulse hard, and about 100. Thirty leeches were ordered to this part of the abdomen, and, when they ceased to bleed, cold rags were to be constantly applied.

* Medico-Chirurgical Notes, by R. FLETCHER, Esq.

In the evening, the pain was much diminished, and the swelling gone. No stools. Sulphate of magnesia every other hour, until the bowels should act. On the third morning, it was reported that he had vomited twice in the night: the belly was swollen more than before, very tender to the touch, and painful. Tongue furred, and pulse 111. He had taken two ounces of the sulphate, and had only one stool. Eighteen ounces of blood were taken from the arm, twelve leeches were applied to the abdomen. A purgative clyster was given: and when the leeches had ceased to bleed, a blister was directed, to cover the whole of the lower portion of the abdomen. He was placed in the warm bath after the leeches dropped off; the bleeding was considerable.

In the evening, he had had ten stools; his belly was much less swollen, he had been vomiting twice, the pain and tenderness much diminished. His pulse, seventy-eight, was reduced to forty-five. Tongue still furred, brown, but not dry. A clyster, with a little infusion of senna and sixty drops of laudanum, was ordered. On the following morning (the sixth from the operation) the swelling and pain of the belly were nearly gone; he had been sick once, had had five stools, pulse seventy-three; slept four hours.

On the fifth day, in the evening, his pulse had risen to eighty-eight, and struck sharp and forcibly against the finger; his tongue was dry, and he was more restless; he had had one stool. Seventeen ounces of blood were taken from his arm; twelve leeches were applied to the epigastric region, where he confessed, upon a close cross examination, that he felt some pain and tenderness. I say cross examined, because it was clear he wished not to be bled any more. He was desired to take sulphate of magnesia every hour until he was purged.

On the sixth morning his pulse was softer, though nearly as frequent; his pain, he declared, was gone; he had had twelve stools; his belly was nowhere tender, tongue more moist.

For the first time, this old man's countenance began to wear rather a haggard look, but his eye was still bright, he spoke firmly, and with confidence that he should recover. He was right. Strength was regained slowly, and he was ultimately discharged cured.

The foregoing case may be interesting, from the extent of the means successfully employed to remove the peritonitis in so aged a person, and from a sacculated stone being left in the bladder with safety.

In four days he lost thirty-five ounces of blood, had fifty-four leeches, and was freely purged; moreover, he endured great pain. But here was a calm, courageous, and somewhat indifferent state of feeling, and if danger was apprehended, little was cared about the result. The patient had not the irritable quickness, the varying and wearing hopes and fears of the sensitive mind, which sees danger in every change, and suffers death many times before its real approach. Had he possessed the latter character, his chance of recovery would have been infinitely less; because the nervous power, which governs the frame, would have been exhausted by its own activity, and those vital actions which depend upon it would, in their turn, have ceased to be continued.

It is well, on this account, for the young practitioner to measure the moral, as well as the physical character of his patients, before he undertakes very decisive means; for, as they are both mixed up together, to form what is called the constitution, this last can never be philosophically known without its component parts being somewhat made out; nor would the practitioner know how much it would bear, were he in ignorance of its true character.

I should not have dared to have taken such bold liberties with this old man's constitution, after it had endured a severe operation, had he possessed this quick, high temperament, sensibility, nervous irritability, or whatever it may be denominated, and which, however it may be compounded of matter or of mind, and in whatever proportions, feels every thing, yields to every thing, leads the body into all possible mischief, and which is rarely remedied, though frequently increased, by bleeding or other lowering means.

I have chosen to consider the peritonitis as the effect of the long-continued and severe sounding previous to the operation, rather than of the latter itself, because there were no circumstances in it which indicated much violence or suffering.

Holiday was well when I last heard of him, and of course the sacculated stone had been in nowise injurious. It is probable, that a calculus, thus closely confined in a sac of the bladder, does little harm there; and when above the pelvis, it is not even likely to congregate more sabulous matter about it. If all this be true, if its close imprisonment admits not of that dreadful rolling of a loose stone in the bottom of the bladder, which irritates, inflames, and thickens the organ, and wears out the patient with pain, why is it thought necessary to ferret out the sacculated one with so much anxiety and positive mischievous violence.

